# Gingival Crevicular Fluid as a Novel Potential Source of Biomarkers Distinguishes Pubertal from Post-Pubertal Subjects

**DOI:** 10.3390/diagnostics6040041

**Published:** 2016-11-17

**Authors:** Xi Wen, Yan Gu, Feng Chen

**Affiliations:** 1 Department of orthodontics, Peking University School and Hospital of Stomatology, No. 22 Zhongguancun Avenue South, Haidian District, Beijing 10081, China; pkuwenxi@163.com; 2 National Engineering Laboratory for Digital and Material Technology of Stomatology, Beijing Key Laboratory of Digital Stomatology, No. 22 Zhongguancun Avenue South, Haidian District, Beijing 10081, China; 3 Central Laboratory, Peking University School and Hospital of Stomatology, No. 22 Zhongguancun Avenue South, Haidian District, Beijing 10081, China

**Keywords:** gingival crevicular fluid (GCF), matrix-assisted laser desorption/ionization time-of-flight mass spectrometry (MALDI-TOF/MS), pubertal growth peak

## Abstract

Detection of pubertal growth peak is vital in orthodontic treatment timing and planning. Gingival crevicular fluid (GCF) contains abundant proteins from different sources and has been proven to be an ideal source of biomarkers. Matrix-assisted laser desorption/ionization time-of-flight mass spectrometry (MALDI-TOF/MS) is an advanced technique that can detect low-molecular-weight peptides with high sensitivity and resolution. The aim of this research was to identify novel candidate biomarkers in GCF to help the diagnosis of pubertal growth peak by MALDI-TOF/MS. Results showed that the peak intensities of six peptides were significantly different between two groups: 1660.2 Da, 1783.0 Da, 2912.5 Da, 4178.6 Da, 5064.9 Da, and 6108.9 Da and are considered to be potential candidate biomarkers to identify pubertal growth peak. Further studies are needed to identify sequence information of these candidate biomarkers.

## 1. Introduction

Accurate identification of different phases of bone maturation plays a vital role in the decision of orthodontic treatment timing in growing patients with skeletal malocclusion. Chronological age and dental age have been proved to be unreliable in identifying growth stage [1,2]. X-ray-based hand-wrist analysis and cervical vertebral maturation (CVM) are the most frequently used methods in clinics. However, these methods are considered to have extra X-ray radiation and not good reproducibility due to the experience of clinicians [3,4]. Thus, stable and noninvasive biomarkers are needed to help the diagnosis of pubertal growth peak. Recent studies showed that new possibilities may be provided by biomarkers in gingival crevicular fluid (GCF) [5,6,7].

GCF is a transudate that flows in the space between healthy free gingiva and root cervix, and the collection procedure is non-invasive and safe [8]. GCF originates mainly from serum and contains abundant proteins from different sources, including host-derived enzymes, degradation products of periodontal tissue, and secreted substances of subgingival bacteria flora [9]. Since its capability of reflecting a host reaction to periodontal inflammation with a site-specific characteristic, GCF has been proven to be an ideal source of biomarkers indicating periodontal diseases [10,11,12] and orthodontic tooth movement [13,14,15]. Some studies have proven that the level of alkaline phosphatase (ALP) in human GCF increased in close relation to a pubertal growth spurt and appears to be a valid source of biomarkers, which provides us with a new possibility of discovering protein biomarkers for pubertal growth spurt [5,6,7].

During the last few decades, mass spectrum-based proteomic analysis has become a focus of research field due to its marked advantages of highly efficiency. A number of studies have applied proteomic analysis with body fluid, such as serum and urine, to investigate candidate biomarkers of systemic diseases [16,17,18]. Matrix-assisted laser desorption/ionization time-of-flight mass spectrometry (MALDI-TOF/MS) is an advanced technique which can detect low-molecular-weight peptides with high sensitivity and resolution [19]. Some studies have applied MALDI-TOF/MS to detect novel biomarkers of periodontitis and successfully discovered several protein biomarkers that have never been investigated before. Ngo et al. conducted MALDI-TOF/MS analysis of GCF and screened for several mass peaks to predict the sites with attachment loss [20]. Luan et al. used MALDI-MS and LC-MALDI-MS/MS of undigested GCF to determine the peptide composition of GCF and successfully detected 33 peptides [21]. However, no studies have employed GCF proteomic analysis to identify novel biomarkers for pubertal growth spurt so far.

Recent studies by Preianò et al. revealed that the processing procedure and storage condition of GCF samples could have a great impact on the GCF profiling by MALDI-TOF/MS [22]. The degradation and modification of proteins in GCF could be a potential source of bias for quantitative measurements and its consequent implications of diagnostic use. Thus, in our study, we used a standard protocol proposed by Preianò et al. in the collection and processing procedure of GCF samples to avoid the potential bias caused by pre-analytical variables [23].

Therefore, the aim of this research was to identify novel candidate biomarkers in GCF to help the diagnosis of pubertal growth peak with the employment of MALDI-TOF/MS, and lay the foundation for future research. Subjects were divided into pubertal and post-pubertal groups based on the CVM method proposed by Baccetti [24], and proteomic analysis of GCF samples was conducted by MALDI-TOF/MS to screen and detect novel biomarkers of pubertal growth.

## 2. Materials and Methods

### 2.1. Ethics Statement

This study was approved by the Peking University Biomedical Ethics Committee (2016-NSF-01, 24 February 2016). All subjects, including adults and parents of pediatric subjects, gave their informed consent for inclusion before participation.

### 2.2. Subjects

Patients seeking orthodontic treatment at Department of Orthodontics, Peking University School of Stomatology between June and August 2016 were recruited for the study. The inclusion and exclusion criteria were as follows: (1) children at pubertal growth spurt or young adults whose wisdom teeth had erupted; (2) periodontal health; (3) had not accepted orthodontic treatment before; (4) in good general health without systematic diseases; (5) no history of trauma; (6) had not taken any medication in the previous 3 months.

All subjects took lateral cephalograms as routine pretreatment examinations and the pubertal growth stage of each volunteer was decided according to the cervical vertebral maturation (CVM) method proposed by Baccetti [24], and the results were confirmed by two clinicians. Forty volunteers were divided into 2 groups based on the different CVM stages. Subjects at CVS 3 and 4 were included in Group I (pubertal group), and subjects at CVS 5 and 6 were included in Group II (post-pubertal group).

### 2.3. Collection of GCF

Before collection of GCF, each volunteers accepted periodontal examinations and those who were detected with attachment loss were excluded. All volunteers accepted ultrasonic scaling a week before GCF collection and were asked to keep a good oral hygiene. The GCF samples were collected from 4 sites of upper central incisors (mediolabial and distolabial) by paper points (DaYaDing, Tianjin, China). Sample sites were isolated by cotton rolls in case of contamination of saliva. Paper points were gently inserted into the gingiva sulcus until a minimum resistance was felt and then left in place for 30 s. Paper points contaminated with blood were discarded. GCF samples were placed in an Eppendorf tube and stored at −80 °C for future analysis.

### 2.4. Sample Preparation

The tips of the four paper points were cut and were visibly wetted by GCF, and the four tips were incubated in 30 μL 2.5% TFA (HPLC grade, MACLIN, Shanghai, China) in the Eppendorf tube. After sonication for 10 min, the sample was centrifuged at 2000× *g* for 10 min at 4 °C. The procedure was repeated for two times, and the elution solution was pooled. The protein concentration was determined using a bicinchoninic acid protein assay kit (CWBio, Beijing, China) and an ELx808 spectrophotometer (BioTek, Hercules, CA, USA).

### 2.5. MALDI-TOF Analysis

The saturated CHCA solution was prepared in 50% water/acetonitrile and 0.1% TFA using a CHCA matrix (Sigma, Georgetown, SC, USA) and sonicated for 1 min. The eluted GCF samples were prepared by a three-step centrifuging method and were mixed with saturated CHCA in a ratio of 1:1. A 1 μL GCF sample and 1 μL of CHCA were spotted on the target plate and allowed to dry at room temperature. MALDI-TOF/MS spectra were acquired in linear mode using the Clin-ToF-II system (BioyongTech, Beijing, China). Spectra with a peptide molecular weight range of 500–10,000 Da were collected using 500 shots of laser energy. The final spectra were obtained by accumulating 100 single signal scans.

### 2.6. Data Processing and Statistics

MS spectra data was processed and analyzed with BioExplorer version 1.0 software (Bioyong Tech, Beijing, China). Processing of mass spectra included smoothing (according to the Savitzky–Golay filters method) and de-nosing (according to discrete wavelet transform). Peaks were detected with the following parameters: S/N: 5; quality factor threshold: 10; minimal intensity: 300. Peak detection was performed on individual spectra. Normalization for peak intensity was performed for statistical analysis. All spectra were normalized to total ion count, and the absolute absorbance intensity was transferred to relative absorbance intensity to reduce the error caused by sample size. The Student’s *t*-test was used to compare the difference in peak intensity between Group I and Group II. A *p*-value smaller than 0.05 (*p* < 0.05) was considered statistical significant.

## 3. Results

### 3.1. Subject Information and MALDI-TOF Spectra

**Figure 1:**
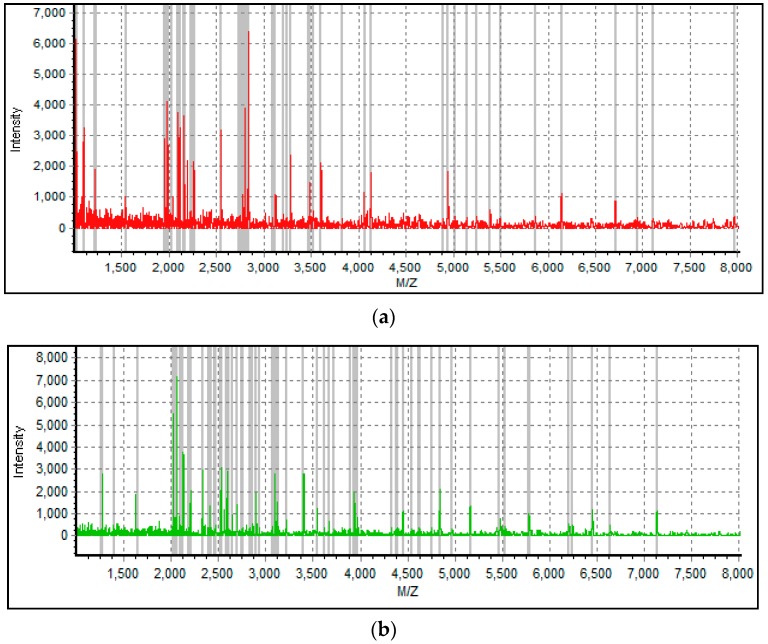
Matrix-assisted laser desorption/ionization time-of-flight mass spectrometry (MALDI-TOF/MS) profiles of gingival crevicular fluid (GCF) from individuals in the pubertal group (**a**) and the post-pubertal group (**b**).

Forty subjects were included in this study, with 20 subjects in Group I and 20 subjects in Group II. Detailed information of the subjects is shown in Table 1. The entire MALDI-TOF mass profile of the GCF samples, in a range from 1000 to 10,000 Da, was obtained (Figure 1). Most mass peaks were detected in 2000–6000 Da.

### 3.2. Statistical Analysis of Mass Peaks

**Figure 2:**
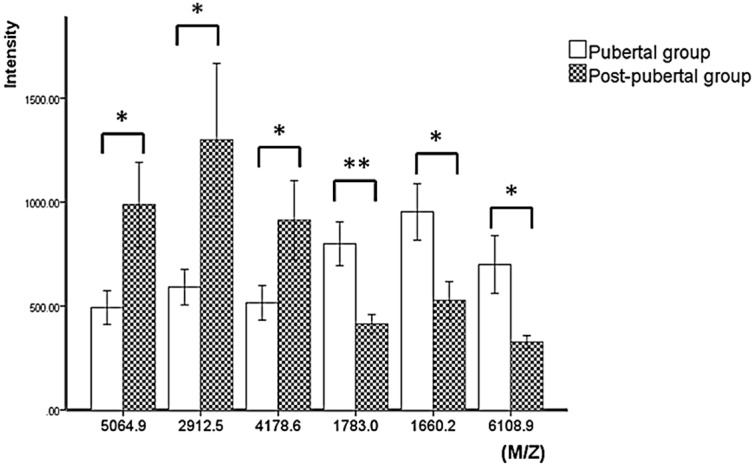
Column view of differential expressed protein peaks between pubertal and post-pubertal groups. Three peptides are upregulated, and three peptides are downregulated from the pubertal group to the post-pubertal group. (* *p* < 0.05, ** *p* < 0.01).

**Figure 3:**
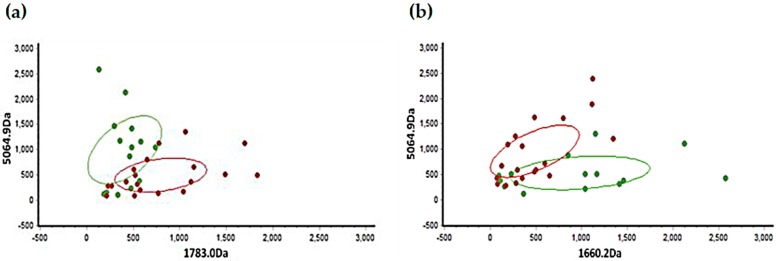
Scatter plots established by the combination of peptides 1783.0 Da and 5064.9 Da (**a**); 1660.2 Da and 5064.9 Da (**b**). Green plots represent pubertal group and red plots represent post-pubertal group.

**Figure 4:**
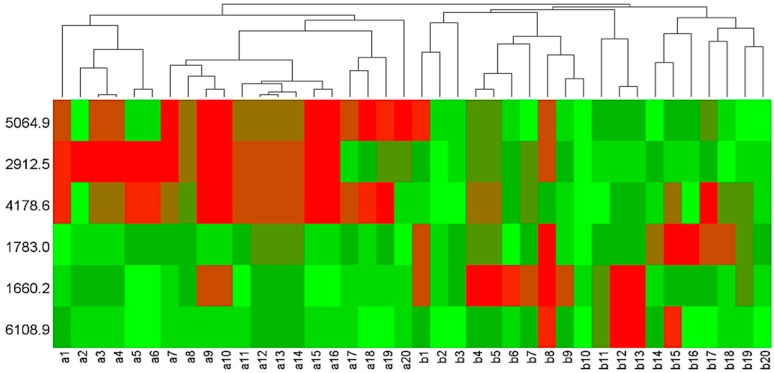
Heat map clustering analysis of six significant mass peaks showed a well-distinguished distribution. (a1–a20 represent post-pubertal group, and b1–b20 represent pubertal group).

An average of 66 peaks were detected in each spectrum. The mass peaks were quantified and compared between the pubertal group and the post-pubertal group. There are six peptides that are significantly different between two groups: 1660.2 Da, 1783.0 Da, 2912.5 Da, 4178.6 Da, 5064.9 Da, and 6108.9 Da, among which three peptides were downregulated, and three peptides were upregulated in the pubertal group (Figure 2). Scatter plots established by a combination of 1783.0 Da and 5064.9 Da (Figure 3a), and 1660.2 Da and 5064.9 Da (Figure 3b), showed a well-distinguished shape (Figure 3). The heat map clustering analysis of six mass peaks showed different distribution between the pubertal and post-pubertal groups (Figure 4).

### 3.3. Validation of Diagnostic Model

A diagnostic model was built using Fisher discrimination analysis and the K-Nearest-Neighbor (KNN) algorithm in BioExplorer software (version 1.0). A threefold cross validation test was carried out, and the results are shown in Table 2. The average accuracy of both models were more than 80%, which was considered to be reliable.

## 4. Discussion

Detection at the stage of pubertal growth is vital in orthodontic treatment timing and planning, especially for subjects with skeletal malocclusion. Cozza et al. found that the amount of supplementary mandibular growth would be significantly larger if the functional treatment is performed at the pubertal peak of skeletal maturation [25]. Perinetti et al. conducted a meta-analysis to compare the treatment effects of removable functional appliance on the pre-pubertal and pubertal Class II patients. Eleven studies were included, and the growth stages of Class II patients were determined by the CVM or HWM methods. Results showed that functional treatment via removable appliance was effective, with a remarkable skeletal effect in correcting Class II malocclusion if it was performed at pubertal stage [26]. Franchi et al. proved that the orthopedic treatment of Class III malocclusion was more effective when it was initiated before pubertal growth spurt [27].

However, the conventional method of identifying growth peaks, including X-ray-based hand-wrist analysis and the CVM method, would cause extra radiation, and reproducibility was believed to be unsatisfied due to the quality of the images and the experience of the clinicians. Gabriel et al. randomly selected 30 individual lateral cephalograms and 30 pairs of lateral cephalograms to test the intraobserver and interobserver agreement of CVM staging among 10 experienced orthodontists. Results showed that the interobserver agreement was below 50% and the intraobserver agreement was only 62%, indicating that the CVM method could not be a strict clinical guideline for orthodontic treatment timing [3]. Nestman et al. considered that the weakness of CVM reproducibility is partly due to the difficulties in classifying the cervical bodies of C3 and C4 as trapezoidal, rectangular horizontal, square, or rectangular vertical [4]. According to research by Zhao et al., a moderate agreement was observed between the gold standard and the CVM method, and the authors suggested that other growth indicators should be taken into consideration in an evaluation of bone maturation [28]. Based on these limitations of CVM methods, we believe that a relatively stable and noninvasive biomarker is needed to help the diagnosis of growth peaks.

Recently, Perinetti et al. found that the ALP activity in GCF of pubertal subjects was significantly higher than that of pre-pubertal and post-pubertal subjects, indicating that the component of GCF would be changed during the pubertal growth and that GCF could be an ideal source of biomarkers for pubertal growth peak [5,6]. GCF is part of saliva that is comprised of complicated protein content and remains more stable regardless of tooth brushing and eating [9], with a characteristic of site-specificity. The total amounts of proteins and peptides that have been identified in GCF in periodontal healthy subjects are reported to be in a range from 88 [29] to 327 [30] that depends on the different sample sizes and methods employed in different studies. Many studies have applied gel-based or gel-free mass spectrometric analysis of GCF proteomic profiling for the detection of novel biomarkers of periodontitis and have successfully discovered several protein biomarkers that have never been investigated before [20,21,31], and some have confirmed the significance of biomarkers that had been proposed in previous studies via conventional research methods [30].

MALDI-TOF/MS is an advanced MS technique and can detect a large range of peptides and proteins with high sensitivity and resolution [19]. In addition, the operation procedure of MALDI-TOF/MS is simple, and the results are easy to interpret, which makes it a useful tool for peptide profile detection. Through MALDI-TOF/MS analysis, we acquired the protein profiles of GCF from subjects in a pubertal group and a post-pubertal group (Figure 1). The intensity and distribution of mass peaks are quite different between the two groups, most of which were in a range of 2000–6000 Da, which was consistent with the result of a previous research by Preianò [23]. In our study, subjects were divided into pubertal and post-pubertal groups based on the CVM method. According to the definition proposed by Baccetti, the subjects at CVS 3 or 4 were included in the pubertal group, and subjects at CVS 5 or 6 were included in the post-pubertal group [24]. As for results, statistical analysis showed that six peptides (1660.2 Da, 1783.0 Da, 2912.5 Da, 4178.6 Da, 5064.9 Da, and 6108.9 Da) were significantly different between the two groups (Table 2, Figure 2). Scatter plots showed that both the combination of 1783.0 Da + 5064.9 Da and 1660.2 Da + 5064.9 Da showed a well-separated shape and could effectively distinguish the pubertal group from the post-pubertal group. Heat map clustering analysis of the six peptides showed a distinct distribution of two groups. These results suggest that the six peptides identified in our study are candidate biomarkers to help the diagnosis of growth peak.

The introduction of the mass spectrum technique into GCF proteomic analysis allows for the identification of multiple candidate biomarkers at a time, which provides an opportunity to propose a statistical model combining several candidate biomarkers to precisely diagnosis diseases. Baliban et al. came up with four different models to predict the presence of peridontitis. Each model contained 10 different biomarkers derived from human and bacteria. Blind test showed that each model reported a sensitivity of 95.2% and a specificity of 100%, and the total prediction accuracy was 97.56% [32], which proves the point that a “biomarker system” is more stable than a single protein, with a more satisfying prediction accuracy.

Based on the results, two diagnostic models were built via different algorithms in BioExplorer software. A cross-validation test was carried out to verify the accuracy of diagnostic models (Table 2). All samples were randomly divided into three sets, two for training and one for validation, and the validation test was carried out three times. Results showed that the average accuracy of both models (Fisher and KNN) were more than 80%, indicating that these two models are both reliable. The diagnostic accuracy of our statistical models was slightly lower than that reported by Baliban [32], possibly due to the smaller sample size we used and the few candidate biomarkers we detected.

However, the analysis of GCF is still challenging for limited volumes (0.2–1.5 μL per site) [21] due to the lack of a standardized protocol for collection and processing [23]. There are still some limitations in our work. The sample size is small, and further studies are needed to reveal the identity of these candidate biomarkers. In subsequent work, we will enlarge the sample size, and use a tandem mass spectrometry (MS/MS) technique to identify the sequence information of these candidate biomarkers. The application of GCF will be expanded and become a promising tool for the diagnosis of growth peak.

## 5. Conclusions

Our research proved via MALDI-TOF/MS that the peptide profile of GCF is different between a pubertal group and a post-pubertal group. Six peptides were significantly different between the two groups—1660.2 Da, 1783.0 Da, 2912.5 Da, 4178.6 Da, 5064.9 Da, and 6108.9 Da—and are considered to be candidate biomarkers of pubertal growth peak. Further studies are needed to identify the sequence information of these candidate biomarkers.

## Figures and Tables

**Table 1:** Detailed information of studied subjects.

**Pubertal Group**			
**Gender**	**Number**	**Mean Age (Years)**	**Mean Concentration (ug/mL)**
Male	10	10.6 ± 1.5	275.3 ± 73.6
Female	10	10.8 ± 1.1	224.8 ± 78.8
**Post-Pubertal Group**			
**Gender**	**Number**	**Mean Age (Years)**	**Mean Concentration (ug/mL)**
Male	10	23.3 ± 0.48	353.4 ± 86.6
Female	10	23.2 ± 0.78	237.8 ± 79.5

**Table 2:** Results of a three-fold cross validation test of two different models.

Models	1	2	3	Average
Fishert	84.62% (11/13)	84.62% (11/13)	85.7% (12/14)	84.98%
KNN	76.92% (10/13)	84.62% (11/13)	85.7% (12/14)	82.41%
